# Foot pain and foot health in an educated population of adults: results from the Glasgow Caledonian University Alumni Foot Health Survey

**DOI:** 10.1186/s13047-018-0290-1

**Published:** 2018-08-17

**Authors:** Gordon J. Hendry, Linda Fenocchi, Jim Woodburn, Martijn Steultjens

**Affiliations:** School of Health & Life Sciences, Glasgow Caledonian University, Cowcaddens Road, Glasgow, G4 0BA Scotland, UK

**Keywords:** Foot pain, Survey, Foot health, Epidemiology

## Abstract

**Background:**

Foot pain is common amongst the general population and impacts negatively on physical function and quality of life. Associations between personal health characteristics, lifestyle/behaviour factors and foot pain have been studied; however, the role of wider determinants of health on foot pain have received relatively little attention. Objectives of this study are i) to describe foot pain and foot health characteristics in an educated population of adults; ii) to explore associations between moderate-to-severe foot pain and a variety of factors including gender, age, medical conditions/co-morbidity/multi-morbidity, key indicators of general health, foot pathologies, and social determinants of health; and iii) to evaluate associations between moderate-to-severe foot pain and foot function, foot health and health-related quality-of-life.

**Methods:**

Between February and March 2018, Glasgow Caledonian University Alumni with a working email address were invited to participate in the cross-sectional electronic survey (anonymously) by email via the Glasgow Caledonian University Alumni Office. The survey was constructed using the REDCap secure web online survey application and sought information on presence/absence of moderate-to-severe foot pain, patient characteristics (age, body mass index, socioeconomic status, occupation class, comorbidities, and foot pathologies). Prevalence data were expressed as absolute frequencies and percentages. Multivariate logistic and linear regressions were undertaken to identify associations 1) between independent variables and moderate-to-severe foot pain, and 2) between moderate-to-severe foot pain and foot function, foot health and health-related quality of life.

**Results:**

Of 50,228 invitations distributed, there were 7707 unique views and 593 valid completions (median age [inter-quartile range] 42 [31–52], 67.3% female) of the survey (7.7% response rate). The sample was comprised predominantly of white Scottish/British (89.4%) working age adults (95%), the majority of whom were overweight or obese (57.9%), and in either full-time or part-time employment (82.5%) as professionals (72.5%). Over two-thirds (68.5%) of the sample were classified in the highest 6 deciles (most affluent) of social deprivation. Moderate-to-severe foot pain affected 236/593 respondents (39.8%). High body mass index, presence of bunions, back pain, rheumatoid arthritis, hip pain and lower occupation class were included in the final multivariate model and all were significantly and independently associated with moderate-to-severe foot pain (*p* < 0.05), except for rheumatoid arthritis (*p* = 0.057). Moderate-to-severe foot pain was significantly and independently associated lower foot function, foot health and health-related quality of life scores following adjustment for age, gender and body mass index (*p* < 0.05).

**Conclusions:**

Moderate-to-severe foot pain was highly prevalent in a university-educated population and was independently associated with female gender, high body mass index, bunions, back pain, hip pain and lower occupational class. Presence of moderate-to-severe foot pain was associated with worse scores for foot function, foot health and health-related quality-of-life. Education attainment does not appear to be protective against moderate-to-severe foot pain.

## Background

Foot pain is reported as common in the general population with prevalence estimates from population surveys ranging from 17 to 30% [1–4]. Several studies have demonstrated associations between foot pain and various factors including increasing age, female gender, higher body mass index (BMI) [5], foot pathologies, footwear habits [6], other musculoskeletal pain [3, 4], and medical conditions including mental health/depression [7], inflammatory arthritis (RA), osteoarthritis (OA), and heart disease [4]. Importantly, foot pain is associated with impaired foot function, disability and health-related quality-of-life [3, 8, 9], and results in significant healthcare system resource use [1, 10]. These studies have contributed to a greater understanding of the populations typically affected by foot pain, the impact of foot pain, and the identification of potentially modifiable risk factors which could be targeted by new foot health management strategies.

Associations between personal health characteristics (such as age, gender), lifestyle/behaviour factors (obesity, footwear) and foot pain have been extensively studied [1, 2, 5, 6, 11]. However, the role of wider determinants of health on foot pain have received relatively little attention in large-scale epidemiological studies of foot pain [12–14]. Only a few studies have studied associations between foot pain and social determinants of health such as lower socioeconomic class, low occupational class, and lower educational attainment [12, 14–16]. This is an important gap in current knowledge as people of lower socioeconomic status tend to experience a higher incidence of a variety health problems, multi-morbidity, pain and disability [17], making their conditions more difficult to manage effectively and increasing the burden of treatment receipt [18, 19]. Indeed, there is strong evidence to suggest that lower socioeconomic status negatively affects treatment outcomes [20], and that treatment burden results in non-adherence and ineffective resource use [21]. Greater understanding of the associations between social determinants of health and foot pain could facilitate the identification of clinically challenging patient groups who may be less likely to respond to contemporary management strategies.

Educational attainment is considered a major social determinant of health which has received relatively little attention in foot pain research. Evidence suggests that more educated individuals generally have fewer comorbidities and are less likely to suffer from long-term diseases, report poor health, and more likely to live longer [22]. This is often termed the education-health gradient [23]. The role of educational attainment in health is complex but it is thought to predict participation in healthy behaviours, avoidance of unhealthy behaviours, less physically demanding occupations and higher income [24]. Education level may be a key correlate of foot pain; whereby lower educational levels may be associated with more severe foot pain. However only a few studies have explored and subsequently demonstrated associations between foot pain and lower educational attainment [14–16, 25]. Therefore, it remains largely unclear as to whether or not educational attainment level plays a protective role against foot pain. Our a priori expectation was that foot pain prevalence would be lower in a university-educated population than the general population. To provide further information, the aims of this study were i) to describe foot pain and foot health characteristics in an educated population of adults; ii) to explore associations between moderate-to-severe foot pain and a variety of factors including gender, age, medical conditions/co-morbidity/multi-morbidity, key indicators of general health, foot pathologies, and social determinants of health; and iii) to evaluate associations between moderate-to-severe foot pain and foot function, foot health and health-related quality of life (HRQoL).

## Methods

The Glasgow Caledonian University (GCU) Alumni Foot Health Survey was a cross-sectional, online (web-based), single-event survey designed to explore foot health characteristics and footwear habits amongst a university-educated population of adults. Ethical approval was obtained from the Glasgow Caledonian University, School of Health and Life Sciences Research Ethics Committee (HLS/PSWAHP/17/152) on the 12th February 2018. Study data were collected and managed using the Research Electronic Data Capture (REDCap) electronic data capture tool hosted at GCU [26]. REDCap is a secure, web-based application designed to support data capture for research studies, providing an intuitive interface for validated data entry. The survey was open for one month between February and March 2018. No reminders were sent during this period.

### Survey administration

Administration of the survey invitation and single-survey-access web-link was undertaken independently of the study team by the GCU Alumni Office in line with data protection principles. Invitations to participate and the web-link to the online survey were distributed via email using Hobsons Radius™ Customer Relationship Management software which permits unique views tracking for robust calculation of survey response rates. Invitees were offered a small incentive to participate (£100 voucher prize draw entry). The opening page of the survey presented the respondent information sheet and consent form to be completed electronically to allow progression to the start of the main survey content.

### Sample selection

The population of interest was GCU Alumni, defined as individuals who have studied at and graduated from GCU, of which there are approximately 140,000 in total since the university was formed in 1993. GCU is a young university (aged 50 years or younger) [27], with three schools including Engineering and Built Environment (subjects related to engineering; computer, communications and interactive systems; and construction and surveying), Business and Society (subjects related to law, economics, accountancy and risk; business management; social sciences; media and journalism), and Health and Life Sciences (subjects related to nursing and community/public health; psychology, social work and allied health sciences; life sciences). This was a large convenience sample who were selected by non-probability sampling.

Eligible respondents for the main survey were those who met all of the following criteria: 1) adults (aged 18 and over), 2) registered as an alumnus with the GCU Alumni Office, 3) registered as resident in the United Kingdom, and 4) had provided a working email address for contact. A total of 50,228 (29,984, 59.7% female) met eligibility criteria and were subsequently sent invitations via email by the GCU Alumni Office.

Based on previous alumni surveys, we estimated that approximately 20% of the 50,228 invitations to be sent to alumni would be viewed by unique individuals (*n* = 10,046), and that approximately 10% of those would respond (*n* = 1005). To enable foot pain prevalence in university educated adults (population of 140,000 total alumni) to be estimated with a margin of error not greater than 5% and confidence limits of a least 95%, a sample size of 384 respondents was required [28, 29].

### Research tool and variables

The web-based anonymous survey and corresponding data dictionary was constructed in REDCap. All responses were by unforced choice. The survey sought demographic and information on respondent characteristics including age (years), gender (male, female, or prefer not to say), employment status (full-time, part-time, voluntary, looking for work, student, looking after home/family, retired, unable to work, other, prefer not to answer), smoking status (smoker, ex-smoker, non-smoker, never smoked, prefer not to say), height and weight, body mass index (BMI) (expressed as a continuous variable and categorical variable for high BMI: ≥25, yes/no) [30]), and ethnicity [31]. Occupation was recorded via free-text entry and allocated to occupation class based on the Standard Occupation Classification 2010 [32] major groups (professional or non-professional occupations). Respondents’ post codes were collected in order to calculate indices of social deprivation [33–35]. These were expressed as lowest 4 deciles (most deprived) versus upper 6 deciles (least deprived).

Medical conditions/comorbidities were evaluated using the Self-Administered Comorbidity Questionnaire (SCQ), a valid and reliable questionnaire [36]. This questionnaire is advantageous over other comorbidity scales as it requires no prior medical knowledge and thus is ideal for self-report [36]. Additional information was sought concerning whether respondents currently suffer from hip and/or knee pain, both of which have previously been associated with foot pain [3, 37].

Self-reported HRQoL was evaluated using the European Quality of Life (Euroqol) EQ-5D-5 L utility index and 100 mm Health visual analogue scale (VAS) (higher scores indicating better health) [38] respectively; which are both extensively validated and widely adopted tools for measuring HRQoL [38–40]. Self-reported physical activity levels were evaluated using the International Physical Activity Questionnaire Short Form (IPAQ-SF), a highly reliable tool with acceptable criterion validity for measurement of physical activity levels [41, 42]. The IPAQ-SF inquires about the number of days and the amount of time spent walking, sitting, or participating in moderate and vigorous-intensity activities [41–43] and provides score categories classifying activity levels as low, moderate or high, which were subsequently dichotomised as low versus moderate-to-high physical activity levels.

**Fig. 1:**
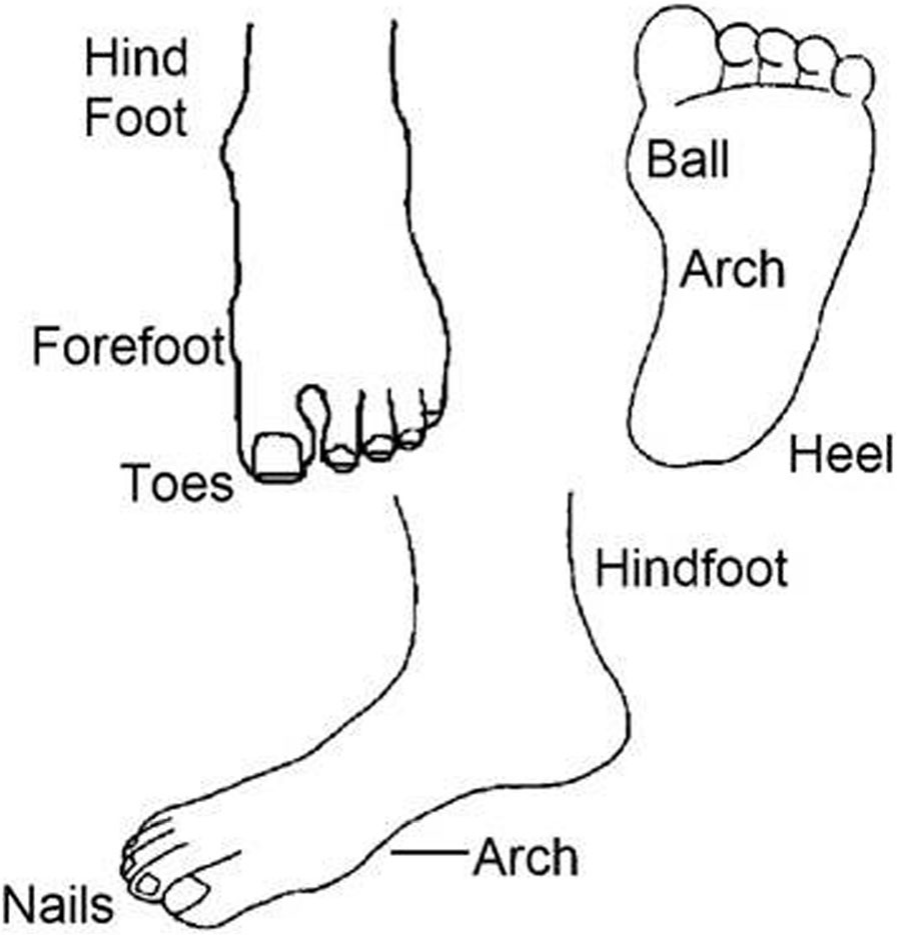
Foot pain map used for self-report of location of foot pain

Current foot pain location (hindfoot, forefoot, toes, ball, arch, heel, nails) was evaluated using a foot pain map (see Fig. 1) previously developed for use in the Framingham Foot Study and frequently adopted in other epidemiological studies of foot pain [3, 44]. Overall foot pain severity was assessed using a 100 mm foot pain VAS scales (0 no pain, 100 worst pain possible) and then categorised as: absent-mild (0–29) and moderate-to-severe (> 30) [45]. Recent history of general foot problems experienced over the previous 6 months was assessed using a check-box list of 7 common foot complaints adopted in previous foot pain surveys [4]. Positive responses to the question concerning bunions generated an additional item within the survey for self-evaluation of hallux valgus severity (mild, moderate and severe); the Manchester Scale for grading of hallux valgus [4], a valid and reliable tool for self-assessment of presence and severity of hallux valgus [46]. Respondents who indicated that they had bunions from the list of 7 common foot complaints were subsequently presented with the four images from the Manchester Scale representing increasing severity of hallux valgus and were asked to self-assess by selecting the image that looked most like their feet. Foot health was evaluated using the Foot Health Status Questionnaire (FHSQ), a valid and reliable 13-item questionnaire with four domains including pain, function, footwear, and general foot health [47, 48].

The survey was pilot tested by six postgraduate students who were independent of the study team and who provided feedback on length, flow, ease of administration, and ease of response [49].

## Statistical analyses

### Survey descriptive analyses

Survey response rates were calculated as a percentage from the total number of unique views of the invitation email after exclusion of invalid responses. Survey data was analysed using descriptive statistics and cross-tabulations, stratified by gender. Where appropriate, continuous data were screened for normality of distributions using the Kolmogorov-Smirnov test. Continuous variables were summarised using medians and inter-quartile ranges (IQR). For categorical data, proportions were calculated and expressed as absolute frequencies (n) and percentages (%).

### Analyses of age, gender and BMI associations

To evaluate whether age, gender and BMI were associated with continuous variables related to foot pain, foot health and HRQoL, univariate linear regression analyses were undertaken. Pearson’s chi-square or Fisher’s exact test statistics with corresponding odds ratios (OR) and 95% confidence intervals (95% CIs) were performed to determine the strength of any significant associations between gender and foot pathologies.

### Univariate analyses for crude associations with moderate-to-severe foot pain

To identify categorical variables with crude associations with moderate-to-severe foot pain, Pearson’s chi-square or Fisher’s exact test statistics with corresponding ORs and 95% CIs were performed. To evaluate whether age (continuous variable) was associated with moderate-to-severe foot pain, univariate logistic regression analysis was undertaken. Candidate variables with a *p*-value of less than 0.2 were added to the multivariate model.

### Multivariate analyses for independent associations with moderate-to-severe foot pain

Associations between moderate-to-severe foot pain (dependent variable) and independent variables identified from crude univariate analyses of association were evaluated using backwards stepwise multivariate logistic regression (p removal 0.10). Hosmer and Lemeshow tests were used to assess goodness-of-fit. Odd ratios and 95% confident intervals are reported as measures of strength of independent associations between independent variables and the dependent variable.

### Analysis of moderate-to-severe foot pain associations with foot function, health and HRQoL

To identify associations between foot function, foot health, HRQoL and moderate-to-severe foot pain, multivariate linear regression analyses were undertaken, with age, gender and BMI entered as known covariates [3]. All tests were two-tailed and *p* values < 0.05 were considered to indicate statistical significance. All analyses were undertaken using IBM® SPSS® version 24.

## Results

Of 50,228 invitations distributed, there were 7707 (15.3%) unique views; from these there were a total of 757 completions of the survey of which 593 (67.3% female; 32.4% male; 0.3% preferred not to say) were valid, giving an overall response rate of 7.7%. There was a higher proportion of women (67.3% v 59.7%) and lower proportion of men (32.4% v 40.3%) amongst responders relative to non-responders respectively. No other information was available for non-responders.

Respondent demographic characteristics are summarised in Table 1. The sample population was comprised predominantly of white Scottish/British (89.4%) working age adults (95%), the majority of whom were overweight or obese (57.9%), and in either full-time or part-time employment (82.5%) as professionals (72.5%). The distribution of age was non-normal, positively skewed, and approximately multimodal for ages 23, 32 and 44 years. Over two-thirds (68.5%) of the sample were classified in the highest 6 deciles (most affluent) of social deprivation.

**Table 1:** Demographics

	Women (*n* = 399)	Men (*n* = 192)	Total (*n* = 593) f
Age, median (IQR) ^a^	42 (31–52)	42 (32–52)	42 (31–52)
BMI, median (IQR) ^b^	25.2 (22.7–29.6)	26.8 (24.0–30.7)	25.8 (23.0–29.9)
Social deprivation (lowest 4 deciles), n (%) ^c^	111 (30.4)	57 (33.5)	169 (31.5)
Professional occupation, n (%)	281 (72.6)	135 (72.6)	416 (72.5)
Employment ^d^			
Paid/self-employed, n (%)	253 (63.9)	137 (72.1)	391 (66.5)
Paid/self-employed, part-time, n (%)	78 (19.7)	16 (8.4)	94 (16.0)
Voluntary work, n (%)	3 (0.8)	5 (2.6)	8 (1.4)
Looking for work, n (%)	10 (2.5)	8 (4.2)	18 (3.1)
Student, n (%)	5 (1.3)	8 (4.2)	13 (2.2)
Looking after the home/family, n (%)	3 (0.8)	1 (0.5)	4 (0.7)
Wholly retired, n (%)	32 (8.1)	12 (6.3)	44 (7.5)
Permanently unable to work, n (%)	2 (0.5)	0	2 (0.3)
Other, n (%)	9 (2.3)	1 (0.5)	10 (1.7)
Prefer not to answer, n (%)	1 (0.3)	2 (1.1)	4 (0.7)
Current smoker, n (%) ^e^	19 (4.8)	13 (6.8)	32 (5.5)
Ethnicity			
White Scottish n (%)	312 (78.2)	147 (76.6)	460 (77.6)
White other British, n (%)	48 (12.0)	22 (11.5)	70 (11.8)
White Irish, n (%)	4 (1.0)	3 (1.6)	7 (1.2)
White Polish, n (%)	5 (1.3)	1 (0.5)	6 (1.0)
Any other white ethnic group, n (%)	9 (2.3)	6 (3.1)	15 (2.5)
Any multiple ethnic groups, n (%)	7 (1.8)	1 (0.5)	8 (1.3)
Pakistani, Pakistani Scottish/British, n (%)	2 (0.5)	2 (1.0)	4 (0.7)
Indian, Indian Scottish/British, n (%)	0	4 (2.1)	6 (1.0)
Chinese, Chinese Scottish/British, n (%)	3 (0.8)	1 (0.5)	4 (0.7)
Any other Asian ethnic group, n (%)	2 (0.5)	0	2 (0.3)
African, African Scottish/British, n (%)	1 (0.3)	3 (1.6)	4 (0.7)
Carribean, Carribean Scottish/British, n (%)	0	1 (0.5)	1 (0.2)
Arab, Arab Scottish/British, n (%)	1 (0.3)	1 (0.5)	2 (0.3)
Any other ethnic group, n (%)	1 (0.3)	0	1 (0.2)
Prefer not to say, n (%)	1 (0.3)	0	2 (0.3)

^a^age n (%) overall, female, male missing values: 15 (2.5); 12 (3.1); 3 (1.6)

^b^BMI n (%) overall, female, male missing values: 47 (7.9); 33 (8.3); 13 (6.8)

^c^Soc dep n (%) overall, female, male missing values: 56 (9.4); 34 (8.8); 22(11.6)

^d^Employment status n (%) overall, female, male missing values: 5 (0.84); 3 (0.78); 2 (1.1)

^e^Smoking status n (%) overall, female, male missing values: 8 (1.3); 6 (1.6); 2 (1.1)

^f^includes 2 cases who preferred not to disclose gender

Foot pain (> 0/100 mm on foot pain VAS scales) experienced in the previous week was common, affecting 331/593 (74%) of respondents and was typically in the mild category (< 30/100 mm). Overall, respondents typically reported sub-optimal foot health for FHSQ subscales relating to pain, general foot health and footwear, but better (near normal) foot function (Table 2).

**Table 2:** Foot pain, foot health, HRQoL characteristics and gender associations

	Women (*n* = 399)	Men (*n* = 192)	Total (*n* = 593) ^d^	Standardised Beta
Foot pain severity (past week) 100 mm VAS scales				
Left foot, median (IQR)	10 (0–45)	8.5 (0–31.8)	10.0 (0–38.5)	0.08
Right foot, median (IQR)	14 (1–50)	4 (0–34.8)	11.0 (0–41.0)	0.08
Overall, median (IQR)	20 (3–53)	13 (0–47.3)	17.0 (2–52.0)	0.09*
Foot Health: FHSQ^a^				
Pain, median (IQR)	72.5 (53.8–84.4)	81.3 (65.6–87.5)	78.1 (59.4–84.4)	− 0.19*
Function, median (IQR)	93.8 (81.3–100.0)	100 (87.5–100.0)	93.8 (81.3–100.0)	− 0.09*
General foot health, median (IQR)	60.0 (42.5–85.0)	72.5 (60.0–85.0)	60.0 (42.5–85.0)	−0.11*
Footwear, median (IQR)	50.0 (25.0–75.0)	75.0 (41.7–100.0)	58.3 (33.3–83.3)	−0.24*
HRQoL: EQ. 5D-5 L				
EQ 5D-5 L Index^b^, median (IQR)	0.837 (0.735–0.879)	0.837 (0.767–1.0)	0.837 (0.740–1.00)	−0.05
EQ 5D-5 L VAS^c^, median (IQR)	82 (70–90)	80 (66–90)	80 (70–90)	0.07

^a^n female = 376; n male = 181; n total 559

^b^n female = 350; n male = 168; n total 520

^c^n female = 342; n male = 163; n total 506

^d^includes 2 cases who preferred not to disclose gender

*univariate linear regression female gender association significant at *p* < 0.05

**Fig. 2:**
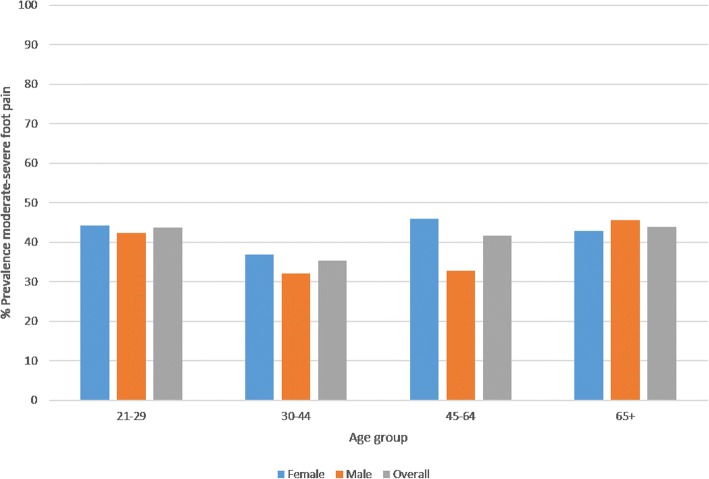
Bar chart representing the lack of a linear relationship between the presence of moderate-to-severe foot pain and increasing age

Female gender was significantly associated with more severe foot pain, worse FHSQ scores for foot pain, foot function, general foot health and footwear subscales (all *p* < 0.05). HRQoL scores were similar for women and men are indicative of suboptimal health states. Age was not associated with foot pain (Fig. 2) or foot function, but was significantly associated with general foot health, footwear-related foot health, and HRQoL (all *p* < 0.05). Higher BMI was significantly associated with poorer foot pain, function, general foot health, footwear-related foot health and HRQoL scores (all *p* < 0.001).

Moderate-to-severe foot pain experienced in the previous week affected 236/593 (prevalence [95% CI] of 39.8%, [35.9–43.7%]). The presence of moderate-to-severe foot pain was not associated with increasing age (OR 1.00, 95% CI 0.99–1.01, *p* = 0.98) or social deprivation (OR 0.86, 95% CI 0.57–1.29, *p* = 0.46), but was associated with non-professional occupation class at the *p* < 0.2 level (OR 1.45, 95% CI 0.99–2.01, *p* = 0.05). Most commonly reported regional sites for pain in the foot were the arch (26.3%), the ball of the foot (24.6%), toes (20.7%), forefoot (20.1%) and the heel (18.5%) (Table 3). Female gender was associated with foot pain affecting the forefoot, ball of the foot, and heel regions (all *p* < 0.05) (Table 4).

**Table 3:** Foot pain, foot health, HRQoL associations with age and BMI

	Standardised beta	*p*-value
Foot pain severity (past week) 100 mm VAS scales		
Age^a^	0.04	0.316
BMI^b^	0.20	< 0.001*
FHSQ Pain		
Age^c^	−0.08	0.051
BMI^d^	− 0.22	< 0.001*
FHSQ Function		
Age^c^	−0.03	0.448
BMI^d^	− 0.29	< 0.001*
FHSQ General foot health		
Age^c^	− 0.13	0.002*
BMI^d^	− 0.25	< 0.001*
FHSQ Footwear		
Age^c^	−0.09	0.040 *
BMI^d^	− 0.17	< 0.001*
EQ 5D-5 L Index^b^, median (IQR)		
Age^e^	− 0.10	0.025*
BMI^f^	− 0.29	< 0.001*
EQ 5D-5 L VAS^c^, median (IQR)		
Age^g^	0.09	0.034 *
BMI^h^	− 0.26	< 0.001*

^a^*n =* 578

^b^*n =* 546

^c^*n =* 544

^d^*n =* 516

^e^*n =* 508

^f^*n =* 483

^g^*n =* 493

^h^*n =* 470

*Multiple linear regression significant at *p* < 0.05

**Table 4:** Regional foot pain characteristics and gender associations

	Women (*n* = 399)	Men (*n* = 192)	Total (*n* = 593) ^a^	Odds ratio (95% CI) F:M
Moderate-to-severe foot pain, n (%)	169 (42.4)	66 (34.4)	236 (39.8)	1.40 (0.98–2.01)
Foot pain region, n (%)				
Hindfoot	47 (11.8)	26 (13.5)	74 (12.5)	0.73 (0.45–1.18)
Unilateral	24 (6.0)	16 (8.3)	40 (6.7)	
Bilateral	23 (5.8)	10 (5.2)	34 (5.7)	
Forefoot	82 (20.6)	37 (19.2)	119 (20.1)	2.24 (1.41–3.56) *
Unilateral	45 (11.3)	26 (13.5)	71 (11.9)	
Bilateral	37 (9.3)	11 (5.7)	48 (8.1)	
Toes	89 (22.3)	34 (18.7)	123 (20.7)	1.42 (0.94–2.15)
Unilateral	45 (11.3)	21 (11.9)	66 (11.1)	
Bilateral	44 (11.0)	13 (6.8)	57 (9.6)	
Ball	102 (25.6)	43 (22.4)	146 (24.6)	1.87 (1.24–2.81) *
Unilateral	41 (10.3)	10 (5.2)	51 (8.6)	
Bilateral	61 (15.3)	33 (17.2)	95 (16.0)	
Arch	105 (26.3)	51 (26.6)	156 (26.3)	1.09 (0.74–1.56)
Unilateral	40 (10.0)	23 (12.0)	63 (10.6)	
Bilateral	65 (16.3)	28 (14.6)	93 (15.7)	
Heel	72 (18.1)	37 (19.3)	110 (18.5)	1.58 (1.01–2.47) *
Unilateral	31 (7.8)	17 (8.9)	49 (8.3)	
Bilateral	41 (10.3)	20 (10.4)	61 (10.3)	
Nails	52 (13)	18 (9.4)	71 (12.0)	1.43 (0.83–2.46)
Unilateral	28 (7.0)	9 (4.7)	37 (6.2)	
Bilateral	24 (6.0)	9 (4.7)	34 (5.7)	

^a^includes 2 cases who preferred not to disclose gender

*Pearson’s chi square significant at *p* < 0.05

Callus and nail problems in the previous 6 months were highly prevalent (Table 5). Female gender was associated with greater prevalence of callous, corns and bunions, and lower prevalence of fungal foot infections (all *p* < 0.05). Respondents that reported having bunions (*n* = 75) subsequently self-assessed these for hallux valgus severity as absent (*n* = 3), mild (*n* = 27), moderate (*n* = 38) and severe (*n* = 7). The presence of bunions was associated with moderate-to-severe foot pain (*p* < 0.05), while the presence of claw toes, and nail problems were associated with moderate-to-severe foot pain at the *p* < 0.2 level.

**Table 5:** Foot pathologies and associations with moderate-to-severe foot pain (MSFP)

	Women (*n* = 399)	Men (*n* = 192)	Odds ratio (95% CI) F:M	Total (*n* = 593)^a^	Odds ratio (95% CI) MSFP vs. no MSFP
Female gender, n (%)	–	–	–	399 (67.5)	1.40 (0.98–2.01)*
Foot problems previous 6 months					
Callous	183 (45.9)	68 (35.4)	1.55 (1.08–2.20)**	252 (42.5)	1.18 (0.73–1.89)
Corns	68 (17.0)	13 (6.8)	2.83 (1.52–5.26)**	81 (13.7)	1.18 (0.73–1.89)
Fungal	58 (14.5)	46 (24.0)	0.54 (0.35–0.83)**	105 (17.5)	1.03 (0.67–1.59)
Verruca	27 (6.8)	7 (3.6)	1.92 (0.82–4.49)	34 (5.7)	1.21 (0.60–2.43)
Bunions	72 (18.0)	3 (1.6)	13.87 (4.31–44.64)**^b^	75 (12.6)	1.66 (1.02–2.69)**
Claw toes	36 (9.0)	15 (7.8)	1.17 (0.62–2.19)	51 (8.6)	1.51 (0.85–2.68)*
Nails	128 (32.1)	58 (30.2)	1.09 (0.75–1.58)	187 (31.5)	1.36 (0.96–1.91)*

^a^includes 2 cases who preferred not to disclose gender

^b^Fisher’s exact test

*significant at *p* < 0.2

**significant at *p* < 0.05

In terms of general health, the majority of respondents reported at least 1 medical condition (55.1%) and approximately one-quarter reported at least 2 medical conditions (multi-morbidity) (26.9%) (Table 6). Three-hundred-and-sixteen (56%) were classed as either overweight or obese despite relatively low proportions being classed as physically inactive. Only 5.5% of respondents were current smokers. Most frequently reported medical conditions/comorbidities were back pain (26.3%), knee pain (23.1%), hip pain (11.8%), depression (11.2%), and high blood pressure (BP) (9.8%) (Table 6); which were all more prevalent amongst female respondents except for high BP. SCQ comorbidity scores were similar for women and men.

**Table 6:** Comorbidities, health characteristics and associations with moderate-to-severe foot pain (MSFP)

	Women (*n* = 399)	Men (*n* = 192)	Total (*n* = 593) ^c^	Odds ratio (95% CI) MSFP vs. no MSFP
Heart disease	4 (1.0)	7 (3.6)	11 (1.9)	0.56 (0.15–2.14)
Treatment	4 (1.0)	5 (2.6)	9 (1.5)	
Limiting	3 (0.8)	0 (0.0)	3 (0.5)	
High BP	36 (9.0)	22 (11.5)	58 (9.8)	1.85 (1.07–3.18) **
Treatment	32 (8.0)	17 (8.9)	49 (8.3)	
Limiting	2 (0.5)	2 (1.0)	4 (0.7)	
Lung Disease	12 (3.0)	4 (2.1)	16 (2.7)	0.91 (0.33–2.5)
Treatment	12 (3.0)	3 (1.6)	15 (2.5)	
Limiting	7 (1.8)	2 (1.0)	9 (1.5)	
Diabetes	8 (2.0)	13 (6.8)	21 (3.5)	0.75 (0.29–1.88
Treatment	6 (1.5)	9 (4.7)	15 (2.5)	
Limiting	0 (0.0)	1 (0.5)	1 (0.2)	
Ulcer/stomach	10 (2.5)	7 (3.6)	17 (2.9)	2.21 (0.83–5.89) *
Treatment	10 (2.5)	5 (2.6)	15 (2.5)	
Limiting	2 (0.5)	1 (0.5)	3 (0.5)	
Kidney disease ^d^	2 (0.5)	3 (1.6)	5 (0.8)	2.29 (0.38–13.78)
Treatment	1 (0.3)	1 (0.5)	2 (0.3)	
Limiting	0 (0.0)	1 (0.5)	1 (0.2)	
Liver disease	0 (0.0)	0 (0.0)	0 (0.0)	–
Treatment	0 (0.0)	0 (0.0)	0 (0.0)	
Limiting	0 (0.0)	0 (0.0)	0 (0.0)	
Anaemia/blood	17 (4.3)	2 (1.0)	19 (3.2)	1.71 (0.69–4.28)
Treatment	14 (3.5)	2 (1.0)	16 (2.7)	
Limiting	2 (0.5)	0 (0.0)	0 (0.0)	
Cancer ^d^	3 (0.8)	2 (1.0)	5 (0.8)	2.29 (0.38–13.78)
Treatment	3 (0.8)	1 (0.5)	4 (0.7)	
Limiting	1 (0.3)	0 (0.0)	0 (0.0)	
Depression	50 (12.5)	17 (8.9)	67 (11.2)	1.54 (0.93–2.57) *
Treatment	41 (10.3)	10 (5.2)	51 (8.6)	
Limiting	25 (6.3)	7 (3.6)	22 (3.7)	
Osteoarthritis	30 (7.5)	3 (1.6)	33 (5.6)	1.88 (0.93–3.81) *
Treatment	14 (3.5)	1 (0.5)	15 (2.5)	
Limiting	19 (4.8)	3 (1.6)	22 (3.7)	
Back pain	114 (28.6)	42 (21.9)	156 (26.3)	2.04 (1.41–2.95) **
Treatment	34 (8.5)	17 (8.9)	51 (8.6)	
Limiting	43 (10.8)	14 (7.3)	57 (9.6)	
Rheumatoid arthritis ^d^	5 (1.3)	4 (2.1)	9 (1.5)	12.49 (1.55–100.54) **
Treatment	5 (1.3)	2 (1.0)	7 (1.2)	
Limiting	1 (0.3)	3 (1.6)	4 (0.7)	
Hip pain	61 (15.3)	9 (4.70)	70 (11.8)	2.72 (1.63–4.55) **
Treatment	17 (4.3)	3 (1.6)	20 (3.4)	
Limiting	28 (7.0)	3 (1.6)	31 (5.2)	
Knee pain	101 (25.3)	36 (18.8)	137 (23.1)	1.56 (1.06–2.30) **
Treatment	30 (7.5)	9 (4.7)	39 (6.6)	
Limiting	54 (13.5)	19 (9.9)	73 (12.3)	
Other	76 (19.0)	39 (20.3)	115 (19.4)	1.50 (0.99–2.27) *
Treatment	38 (9.5)	18 (9.4)	56 (9.4)	
Limiting	15 (3.8)	15 (7.8)	30 (5.1)	
SCQ total, median (IQR)	1 (0–3)	1 (0–3)	1 (0–3)	–
≥ 1 Medical condition	226 (56.6)	101 (52.6)	327 (55.1)	1.94 (1.38–2.72) **
≥ 2 Medical conditions (multi-morbidity)	110 (27.6)	50 (26.0)	160 (26.9)	2.04 (1.42–2.95) **
Overweight ^a^	193 (52.7)	122 (63.5)	316 (56.0)	2.01 (1.41–2.88) **
Low physical activity ^b^	45 (11.3)	18 (9.4)	64 (12.5)	0.93 (0.54–1.58)
Current smoker	19 (4.8)	13 (6.8)	32 (5.4)	0.90 (0.43–1.88)

^a^*n* = 47 values missing

^b^*n* = 82 values missing

^c^includes 2 cases who preferred not to disclose gender

^d^Fisher’s exact test conducted where cell frequencies < 5

*significant at *p* < 0.2

**significant at *p* < 0.05

**Fig. 3:**
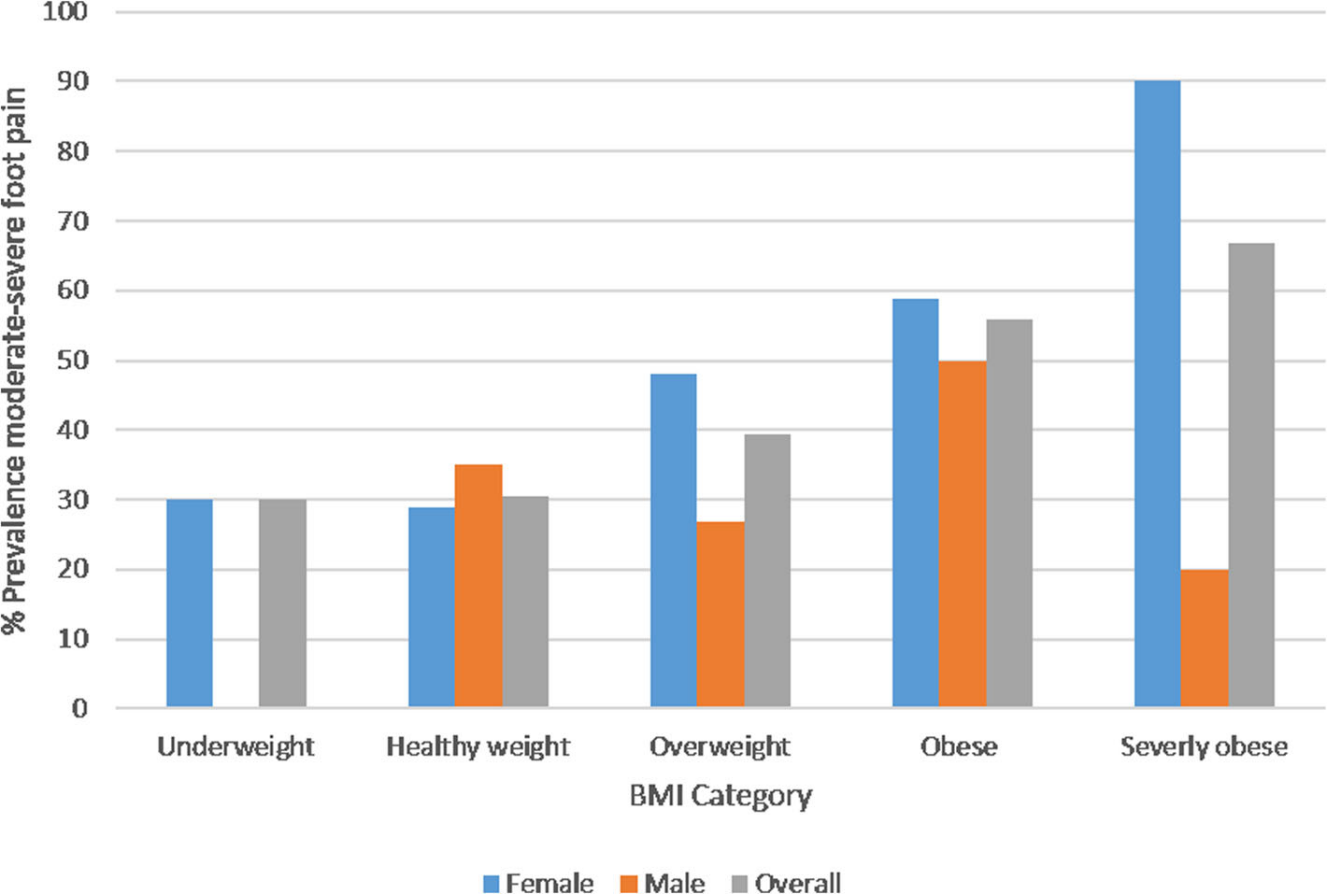
Bar chart depicting the increasing prevalence of moderate-to-severe foot pain with increasing BMI

Medical conditions back pain, knee pain, hip pain, high BP, rheumatoid arthritis, being overweight/obese (see Fig. 3) and having either any one medical condition or multi-morbidity were all associated with moderate-to-severe foot pain (*p* < 0.05). Ulcer/stomach disease, depression, OA and ‘other’ comorbidities were associated with moderate-to-severe foot pain at the *p* < 0.2 level.

Following univariate analyses, categorical variables gender, and presence/absence of bunions, claw toes, nail problems, high BP, ulcer/stomach disease, depression, OA, back pain, RA, hip pain, knee pain, one or more medical conditions (comorbidity), high BMI (> 25), and occupation class (non-professional) were included in the stepwise multivariate model. High BMI, bunions, back pain, RA, hip pain and occupation class were included in the final model (Table 7) and all were significantly and independently associated with moderate-to-severe foot pain (*p* < 0.05) except for RA (*p* = 0.057). This model was a statistically adequate fit to the observed data (Hosmer and Lemeshow *p* = 0.366).

**Table 7:** Multivariate logistic regression model for independent associations between independent variables and moderate-to-severe foot pain

Variable	B	SE	Wald	*p*-value	OR (95% CI)
Bunions	0.63	0.28	5.25	0.022*	1.88 (1.09–3.21)
Back pain	0.64	0.21	9.22	0.002*	1.90 (1.26–2.88)
RA	1.74	1.11	2.44	0.118	5.67 (0.64–50.02)
Hip Pain	0.68	0.31	4.96	0.026*	1.97 (1.09–3.59)
BMI Overweight	0.61	0.19	10.06	0.002*	1.84 (1.26–2.68)
Occupation Class	0.45	0.21	4.53	0.033*	1.57 (1.04–2.37)
Constant	−1.279	0.18	52.70	0.000*	
Model performance			χ^2^, df		
Hosmer Lemeshow	–	–	6.46, 6	0.373	–

*N* = 530 included in multivariate model

Respondents with moderate-to-severe foot pain had significantly worse scores for foot function, foot health and HRQoL compared to those without (Table 8).

**Table 8:** Multivariate linear regression models for associations between moderate-to-severe-foot pain, foot function, foot health and HRQoL

	No MSFP	Yes MSFP
FHSQ Foot Function ^a^, median (IQR)*	100 (93.75–100)	87.5 (68.75–93.75)
FHSQ General Foot Health ^a^, median (IQR)*	85 (60–85)	60 (25–72)
EQ 5D-5 L Index ^b^, median (IQR)*	84 (75–90)	76 (60–87)
EQ 5D-5 L VAS ^c^, median (IQR)*	0.85 (0.768–1.00)	0.767 (0.697–0.837)

^a^*n* = 559

^b^*n* = 506

^c^*n* = 520

*significance at *p* < 0.05 adjusted for age, gender and BMI

## Discussion

Despite recruiting a large sample size which met the minimum target required for estimation of foot pain prevalence, this survey achieved a response rate of 7.7% which was lower than a priori expectations. Little information was available to permit robust responder versus non-responder comparisons; however, for available data, a difference was observed for gender with proportionally more women amongst responders relative to non-responders. While cautious interpretation is warranted due to potential non-response bias, tentatively these findings may be generalizable to other university educated populations who graduated from young universities offering similar prospectuses, with similar student demographic profiles. The true size of the target population is unknown; however, Labour Force Survey data suggests that 42% of the United Kingdom (UK) population aged 21 to 64 in 2017 had achieved higher education qualifications [50].

The results of this survey demonstrate that moderate-to-severe foot pain was highly prevalent in a university educated population of adults, affecting approximately 40% of respondents, and was associated with poorer foot health and reduced HRQoL. While definitions vary, the prevalence of foot pain reported in this study is significantly higher than estimates reported in population surveys [1, 2, 4]. This could be explained in part by the general health characteristics of the respondents amongst whom there was high prevalence of obesity, comorbidity, and multi-morbidity. Previous studies have identified that both risk factors and pathogenic links between musculoskeletal disorders and other medical conditions often result in development multi-morbidity; for example, obesity is a known risk factor for both OA and diabetes [51]. While the general health characteristics were typically poor given the age range of the sample, it is possible that the high foot pain prevalence estimates were vulnerable to non-response bias, whereby respondents with the condition of interest (foot pain) were more likely to respond.

The high prevalence of foot pain, comorbidity and multi-morbidity in this sample may also be explained by the student recruitment profile of GCU and the demographic characteristics of its students. One of GCU’s main goals is to widen access to higher education and 17% of new entrants in 2017 were from the lowest quintile of social deprivation, higher than the sector average of 10% [52]. At the GCU Glasgow campus more than 70% of students come from Greater Glasgow or the West of Scotland and around 10% are from the most deprived areas in Scotland. The socioeconomic composition of Glasgow differs to that of the rest of Scotland and the UK, with higher levels of deprivation, poor health behaviours, disease, suicide and lower life expectancy [53–55]. Interestingly, at the time of the survey respondents were largely from upper 6 deciles (more affluent) for social deprivation, and predominantly employed in high-level managerial or professional occupations. However, a major limitation of using current measures of social deprivation is that they do not necessarily account for cumulative exposures to social deprivation experienced earlier in life [56].

A strength of the current survey is that the correlates of foot pain identified appear to be largely theoretically consistent with findings from other large-scale population surveys [3, 4]. Foot pain and foot health characteristics were generally worse amongst females, with female gender associated with greater prevalence of pain affecting heel, forefoot and ball of the foot [3, 4, 57]. Moreover, presence of superficial foot problems such as callous, corns and nails problems, as well as structural problems such as bunions were associated with female gender, whilst the presence of fungal infections was associated with male gender [4]. Similar findings have been previously attributed to relatively poor footwear habits adopted by women compared to men [3, 6]. Future analyses will seek to evaluate the role of past and current footwear habits on foot pain and foot health characteristics in this sample.

This study is in agreement with previous studies in that poorer foot health, footwear-related foot health and HRQoL were all significantly associated with increasing age [8, 58]. A somewhat surprising finding was that age was not significantly associated with foot pain severity. Less than 5% of respondents were over the age of 65, and of those 44% had moderate-to-severe foot pain. Whilst this prevalence estimate is higher than has been reported previously for the over 65 age range in other studies [3, 4]; foot pain prevalence was generally high across all age groups with a u-shaped distribution ranging from 30 to 44%, instead of increasing linearly with age. Given the nature of the sampling frame targeting alumni of a young university, a proportional representation of < 5% within the sample in the over 65 years’ age category was not an unexpected finding and therefore these findings do not appear to be due to under-representation of this age group.

Multivariate analyses identified that female gender, obesity, bunions, hip pain, back pain and lower occupational class were significantly and independently associated with moderate-to-severe foot pain. These findings are consistent with previous studies [3, 4, 14]. Obesity has been identified as a significant predictor of non-specific foot pain in several studies and has been linked to increased stresses applied to the foot and metabolic pathways [59–63]. Recent research has demonstrated that weight loss (via bariatric surgery) results in reductions in foot pain severity, suggesting that weight-loss strategies may be a credible therapeutic option for managing foot pain [64]. The role of physical activity in weight-loss strategies to improve health has been well established [65]. However, adherence to weight-loss strategies particularly in the presence of pain is difficult for many people and strategies to promote maintenance of physical activity can be challenging to implement [66]. Brief interventions for physical activity delivered in patient consultation have received attention recently but little evidence is available for their long-term effectiveness [67, 68].

The presence of self-reported bunions was associated with moderate-to-severe pain, and was significantly more prevalent in women, which was in agreement with previous studies [69–71]. The term “bunion” is a lay term often used by members of the general public to describe a medial swelling affecting the first metatarsophalangeal joint. We acknowledge that this term not necessarily interchangeable or synonymous with “hallux valgus” which is characterised by abnormal rotation and lateral deviation of the big toe and as such these results should be interpreted with caution. The lay-friendly term “bunion” was included in the survey to allow self-report of big toe pathology which may or may not have been a true hallux valgus if assessed via clinical examination. This was supplemented with generation of an additional survey item for self-assessment of hallux valgus severity [46] which was graded as at least mild in 72 of 75 respondents who self-reported bunions. With cautious interpretation, the prevalence of self-reported bunions was comparable to other population studies reporting hallux valgus prevalence and has been associated with impaired function, reduced HRQoL and an increased risk of falls in older adults [72]. Conservative management of hallux valgus is predominantly aimed at reducing the need for or delaying surgical intervention, and involves use of foot orthoses, rocker-sole footwear and/or physical therapies to improve first ray function and reduce pain [73, 74]. Known risk factors for hallux valgus are mostly non-modifiable such as female gender and foot structure (i.e. dorsiflexed first metatarsal), however high BMI and poor footwear habits have been identified as modifiable risk factors [70]. Recent evidence suggests that improvements in footwear habits and outcomes related to foot pain and foot function in women aged 50 or over can be achieved with standardised advice on proper shoe characteristics and fit [75, 76]. It is unclear whether similar outcomes could be replicated in a younger cohort of women who may have different footwear preferences [77, 78].

Back pain and hip pain were independently associated with moderate-to-severe foot pain and this finding is in agreement with a large body of evidence demonstrating strong associations between foot pain and other regional bodily pain [3, 4, 37]. Multi-site joint problems have been identified as common and are associated with increasing age, the presence of comorbidities and social deprivation [37, 79]. Recent evidence suggests that multi-site joint pain prevalence is increasing and that more holistic management strategies are required whereby health professionals consider joint pain beyond the primary site [79]. This might provide an implementation challenge for podiatrists who are trained in the assessment and management of lower limb complaints, and further referrals to other health professionals such as physiotherapists might add to patients’ treatment burden in the context of multimorbidity [18].

Non-professional occupation class was independently associated with of moderate-to-severe foot pain. This confirms the findings from previous research suggesting that a lower class of occupation, which include manual occupations, is a known risk factor for pain and disability [12, 14, 15]. This was an interesting finding given that social deprivation measured via indices of social deprivation was not found to be associated with foot pain. Use of occupation class as a proxy measure of socioeconomic status is advantageous as it is associated with other social determinants of health including social status, level of income and level of education attainment [80]. Recent research has shown that a life-course of consistently low socioeconomic position results in a significantly greater odds of reporting chronic disabling pain [81]. Given that this sample all achieved a university level education, it is possible that occupation class is a more useful indicator of cumulative socioeconomic status in this cross-sectional study context than area-based indices of social deprivation, which are vulnerable to the ecological fallacy [80]. These results suggest that while a high level of education attainment may not be protective against moderate-to-severe foot pain, a higher level of occupation class may be. It is possible that this is due to the greater physical demand associated with lower class occupations [15].

There are some limitations to this study that warrant further attention. The response rate was lower than anticipated and little information was available to allow non-responders analyses and so was vulnerable to non-response bias. A small incentive (£100 prize draw entry) was offered to participants to boost recruitment rates but this may have resulted in a reduction in data quality, whereby respondents increased satisficing behaviours such as item skipping, rushing, and straight-lining (the act of selecting the same response over and over again for fast completion of a survey) [82, 83]. The survey was administered and completed electronically and required invitees to have an up to date and working email address. This may have resulted in selection bias since not all of the target population would have been able to receive the survey invitation [84]. The sampling frame restricting eligibility to alumni from a single university in Glasgow, with its known health issues, limits the ability to generalizability the survey findings to other university educated populations in the UK. Given the focus on a pre-specified subgroup, these results are not generalizable to the general population. However previous alumni studies such as the Harvard Alumni Study [85] have highlighted that narrow eligibility criteria can be advantageous in terms of convenience for recruiting large samples and achieving statistical power for analyses of group with specific demographic characteristics (such as education level attainment). The survey was cross-sectional in nature and therefore do not reflect causality nor patterns of foot pain and health status beyond a single discrete point in time. Lastly, responses in relation to foot problems and pathologies were by self-report, which may have resulted in some measurement error due to lack of prior knowledge about these conditions amongst respondents.

## Conclusions

Moderate-to-severe foot pain was highly prevalent in a university-educated population and was independently associated with female gender, bunions, obesity, back pain, hip pain and lower occupational class. Respondents with moderate-to-severe foot pain had significantly worse scores for foot function, foot health and HRQoL. Education attainment does not appear to be protective against moderate-to-severe foot pain.

## Abbreviations

BMI: Body mass index
BP: Blood pressure
CI: Confidence interval
EQ 5D-5 L: Euro quality of life (Euroqol) five dimension 5 level questionnaire
FHSQ: Foot health status questionnaire
GCU: Glasgow Caledonian University
HRQoL: Health-related quality of life
IPAQ-SF: International physical activity questionnaire short form
IQR: Inter-quartile range
OA: Osteoarthritis
OR: Odds ratio
RA: Rheumatoid arthritis
REDCap: Research Electronic Data Capture
SCQ: Self-assessment comorbidity questionnaire
UK: United Kingdom
VAS: Visual analogue scale
